# Foxp3^+^Helios^+^ regulatory T cells are associated with monocyte subsets and their PD-1 expression during acute HIV-1 infection

**DOI:** 10.1186/s12865-019-0319-7

**Published:** 2019-10-24

**Authors:** Lifeng Liu, Qiuyue Zhang, Peng Chen, Na Guo, Aixin Song, Xiaojie Huang, Wei Xia, Li Li, Christiane Moog, Hao Wu, Bin Su, Tong Zhang

**Affiliations:** 1 Center for Infectious Diseases, Beijing Youan Hospital, Capital Medical University, Beijing, 100069 China; 2 Beijing Key Laboratory for HIV/AIDS Research, Beijing, 100069 China; 3 INSERM U1109, Fédération de Médecine Translationnelle de Strasbourg (FMTS), Université de Strasbourg, 67000 Strasbourg, France

**Keywords:** Foxp3^+^Helios^+^, Monocyte subsets, PD-1, Acute HIV-1 infection

## Abstract

**Background:**

Helios has been reported to stabilize regulatory T (Treg) suppressive function. Programmed cell death protein 1 (PD-1) expression in three human monocyte subsets modulates immune responses. Recently, our team reported that three monocyte subsets are associated with T helper cell differentiation in HIV-1-infected patients. Until now, the effects of monocyte subsets and their PD-1 expression on Foxp3^+^Helios^+^ Treg cells have not been fully characterized, especially during acute HIV-1 infection.

**Results:**

The frequency of Foxp3^+^Helios^+^CD45RA^+^ Treg cells is significantly higher in patients with acute HIV-1 infection than those of healthy controls and chronic HIV-1-infected patients undergoing combined antiretroviral therapy. The frequency of Foxp3^+^Helios^+^CD45RA^+^ Treg cells is inversely correlated with CD4 T-cell counts and the CD4/CD8 ratio in chronic HIV-1-infected patients. During acute HIV-1 infection, the frequency of Foxp3^+^Helios^+^CD45RA^+^ Treg cells is inversely correlated with the frequency of the intermediate CD14^++^CD16^+^ monocyte subset, but positively correlated with PD-1 expression in both intermediate CD14^++^CD16^+^ and non-classical CD14^+^CD16^++^ monocyte subsets.

**Conclusions:**

In this study, the perturbations of Foxp3^+^Helios^+^ Treg cells were characterized, and the association between monocyte subsets and their PD-1 expression and Foxp3^+^Helios^+^ Treg cells was evaluated during HIV-1 infection. Our observations provide new evidence of the roles for Foxp3^+^Helios^+^ Treg cells and PD-1 expression on monocyte subsets in HIV pathogenesis.

## Background

Foxp3^+^ regulatory T (Treg) cells play a pivotal role in the regulation of the immune response due to suppressive abilities. The frequency of Tregs was associated with an inadequate immunological response during HIV-1 infection [1]. Natural regulatory T (nTreg) cells are produced in the thymus, while inducible regulatory T (iTreg) cells or adaptive Treg cells can be induced in vitro in the presence of IL-2 and TGF-β [2]. Compared with Foxp3^+^Helios^+^, the traditional combination of CD25 and Foxp3 surface markers underestimates the proportion of Treg cells [3]. Helios, one of the Ikaros zinc finger transcription factors, was reported to bind to the Foxp3 promoter and stabilize the suppressive function of Treg cells [4]. In the periphery, Helios is expressed in approximately 70% of CD4^+^Foxp3^+^ human and mouse Treg cells [5]. In HIV-1-infected individuals who were on combined antiretroviral therapy (cART), the frequency of memory Foxp3^+^Helios^+^ Treg cells was significantly higher than that of healthy controls, whereas the frequency of memory Foxp3^+^Helios^−^ Treg cells was similar to that of healthy controls [6]. Until now, Helios expression in T-regulatory cells has not been fully characterized in HIV-1-infected patients, especially during acute HIV-1 infection (AHI).

Monocytes can act as regulators of inflammation and HIV-related comorbidities [7]. Monocytes are heterogeneous with different phenotypes and functions, and in 2010, the following three monocyte subsets were recommended by the Nomenclature Committee of the International Union of Immunological Societies: classical (CD14^++^CD16^−^), intermediate (CD14^++^CD16^+^), and non-classical (CD14^+^CD16^++^) subsets [8]. Monocytes have many immunological functions, including antigen presentation, making them a link between the innate and adaptive immune systems [9]. Monocytes were reported to control Treg cell differentiation. Activated monocytes can influence Treg cells through the production of soluble mediators, CD16^+^ monocytes inhibit proliferation of Helios^+^ Tregs through IL-12, whereas Helios^−^ Tregs are suppressed by CD16^−^ monocytes via TNF-α [10]. The specific roles of three monocyte subsets on Treg cell differentiation have remained poorly understood in HIV-1-infected patients.

Programmed cell death protein 1 (PD-1) can be expressed on T cells, B cells and other cell types. PD-1 expression on T cells and its binding by PD ligand 1 (PD-L1) on antigen-presenting cells (APCs) is a major mechanism that results in T-cell exhaustion [11–13]. PD-1 signaling may also occur independently of T-cell or B-cell antigen receptor signaling [14]. PD-1 can be induced and expressed on human monocytes during HIV-1 infection [15]. The triggering of PD-1 on monocytes was shown to induce IL-10 production and inhibit CD4 T-cell proliferation, which indicates that PD-1 on monocytes could modulate immune responses by inducing IL-10 production [16]. The therapeutic potential of manipulating PD-1 and PD-1 ligands provides an impetus to understand the function of PD-1 expression on APCs [17]. Limited data are available on PD-1 expression on three monocyte subsets and their association with Treg cells during HIV-1 infection.

Host immune responses during AHI can influence the establishment of the viral setpoint, which is a predictor of disease progression [18]. We previously found that in acute HIV-1-infected patients, the frequency of the intermediate CD14^++^CD16^+^ monocyte subsets was positively associated with the frequency of IL-4, whereas this subset was positively associated with the frequency of IFN-γ and IL-4 producing CD4^+^ T cells in chronic HIV-1-infected cART-naïve patients [19]. In the present study, we explored Helios expression in Treg cells in HIV-1-infected patients, and we evaluated the association between Foxp3^+^Helios^+^ Treg cells and the levels of three monocyte subsets and their PD-1 expression.

## Methods

### Study participants

One hundred and thirty-two participants were enrolled in this study, and written informed consent was provided according to the Declaration of Helsinki. These individuals were classified into four groups: thirty-six men who had sex with men (MSM) with acute HIV-1 infection (AHI), twenty-eight chronic HIV-1-infected cART-naïve patients (CHI&ART-), thirty-two chronic HIV-1-infected patients with undetectable viral load after cART (CHI&ART+), and thirty-six male healthy controls (HC). The inclusion and exclusion criteria for each group were the same as previously described [19].

### Cell surface and intracellular cytokine staining

Cell surface and intracellular cytokine staining were performed as previously described [20]. Monocyte phenotypes and Foxp3^+^Helios^+^ Treg cells were stained with the following antibodies: anti-CD14-FITC (eBioscience), anti-CD16-PE (eBioscience), anti-HLA-DR-eFluor® 450 (eBioscience), anti-human CD279 (PD-1)-PerCP-eFluor® 710 (eBioscience); anti-CD4-PE-Cy7 (eBioscience), anti-xhuman CD45RO-APC-eFluor® 780 (eBioscience), anti-human CD45RA-eFluor® 450 (eBioscience), and anti-human CD25-PE-Cy7 (eBioscience) anti-Foxp3-PE (eBioscience), and anti-Helios APC (eBioscience Inc. San Diego CA).

### CD4^+^ T-cell count and viral load measurements

The CD4^+^ T-cell count was determined by three-color flow cytometry using CD3-APC, CD4-FITC, and CD8-PE monoclonal antibodies (BD Biosciences). Then, an analysis was conducted using the BD FACS Canto™ II Flow cytometry system (BD Biosciences, San Jose, CA). HIV-1 viral load tests were performed using an automated real-time PCR-based *m*2000 system (Abbott Molecular Inc., Des Plaines, IL) according to the manufacturers’ instructions.

### Statistics

Statistical analyses were performed using an ANOVA test, Student’s t-test or non-parametric tests. Spearman’s rank-order nonparametric correlation test was used to analyze the relationship between two variables. Statistical analyses were performed with GraphPad Prism software version 5.03 (GraphPad Software, San Diego, California, USA). All reported *p* values were two-tailed and considered significant at *p* < 0.05(*) or *p* < 0.01(**).

## Results

### Characteristics of participants

Thirty-six MSM with AHI, 28 CHI&ART- patients, 32 CHI&ART+ patients, and 36 HCs were enrolled in the study. Parameters, including age, viral load, cell counts and CD4^+^/CD8^+^ ratio, are presented in Table 1. The ages between the HIV-1-infected patients and HC were matched. The mean CD4 T-cell counts and CD4/CD8 ratios of HC were higher than those of the acute HIV-1-infected patients (*p* < 0.01) and higher than those of chronic HIV-1-infected patients without cART (*p* < 0.01).

**Table 1:** Characteristics of HIV-1-infected patients and healthy controls

	Healthy controls	Acute HIV-1-infected patients	Chronic cART-naïve HIV-1-infected patients	Chronic HIV-1-infected patients with cART
Cases, no	36	36	28	32
Age (years)	28.3 (21–46)	29.5 (20–48)	28.7 (20–47)	29.2 (21–46)
HIV-RNA (copies/ml)	NA	316,924.7 (2409–6,313,483)	26,934.1 (567–147,548)	TND
CD4 (cells/μl)	748 (328–1506)	470 (215–762)	311 (134–638)	672 (318–1280)
CD4/CD8 ratio	1.13 (0.43–2.16)	0.49 (0.1–1.13)	0.33 (0.11–0.68)	0.75 (0.29–1.57)

Data are presented as the means with ranges

Abbreviations: *cART* combined antiretroviral therapy, *NA* not available, *TND* target not detected

### Helios expression in T-regulatory cells in HIV-1-infected individuals

**Fig. 1:**
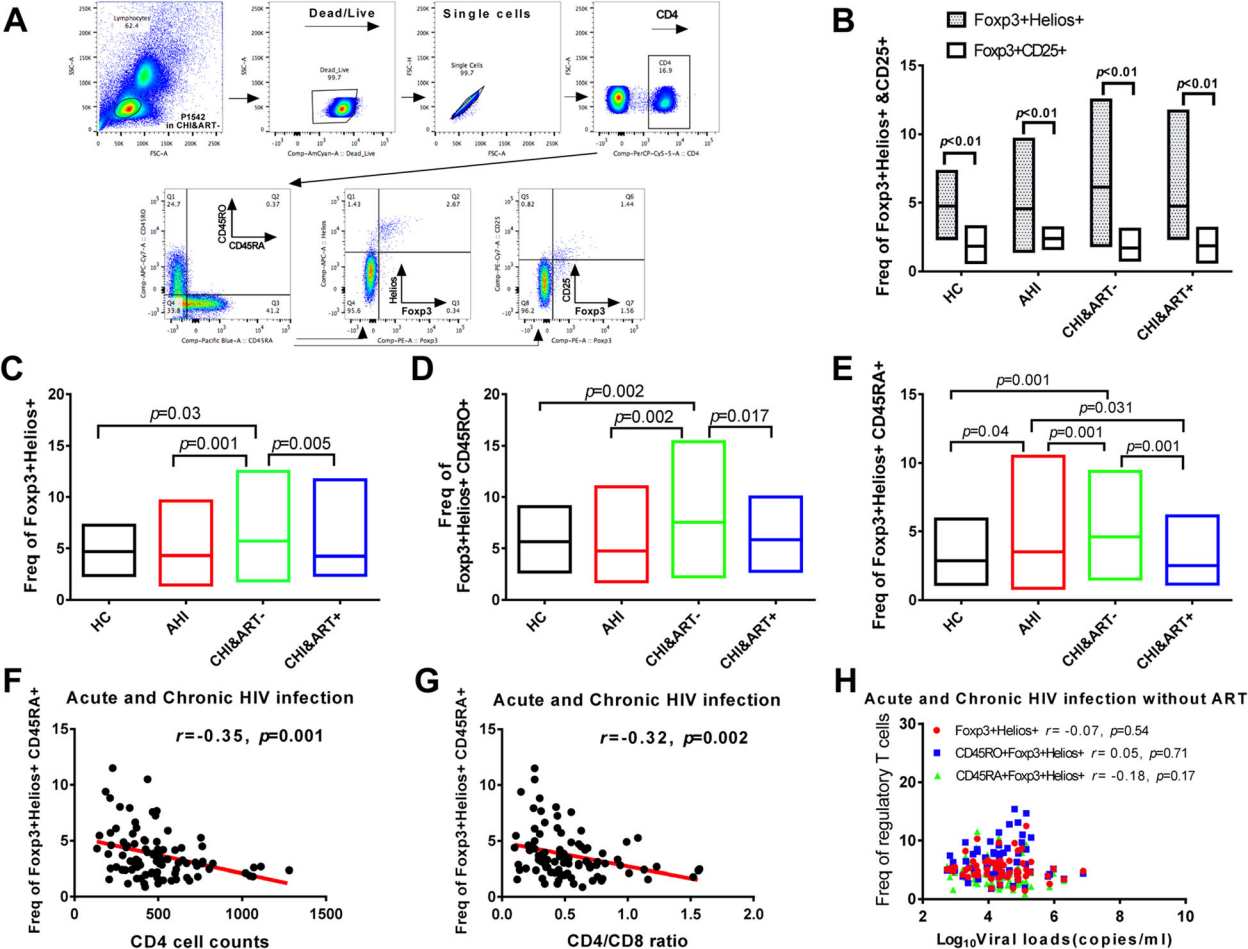
Perturbations of Foxp3^+^Helios^+^ Treg cells in HIV-1-infected individuals. The gating strategy for analysis of Foxp3^+^Helios^+^ and Foxp3^+^CD25^+^ is indicated (**a**). Paired comparison of %Foxp3^+^CD25^+^ (blank) and %Foxp3^+^Helios^+^ (gray) Treg populations in HIV-1-infected patients (**b**). Frequencies of Foxp3^+^Helios^+^ (**c**), Foxp3^+^Helios^+^CD45RO^+^ (**d**), and Foxp3^+^Helios^+^CD45RA^+^ (**e**) were determined by flow cytometry in HC, AHI, CHI&ART-, and CHI&ART+ patients. The box in Fig. 1 marks the min and max values, and the horizontal lines in (**c**), (**d**), and (**e**) depict median values. Correlation between the frequency of Foxp3^+^Helios^+^CD45RA^+^ and CD4 T-cell counts (**f**) as well as the CD4/CD8 ratio (**g**) during acute and chronic HIV-1 infection. Correlation between viral loads and the levels of Foxp3^+^Helios^+^ (circular), Foxp3^+^Helios^+^CD45RO^+^ (square), and Foxp3^+^Helios^+^CD45RA^+^ Treg cells (triangle) in AHI and CHI&ART- patients (**h**). All *p* values were calculated using an ANOVA, Student’s t-test or Mann-Whitney U test, and the Spearman correlation test

The perturbations of Foxp3^+^Helios^+^ Treg cells are shown in Fig. 1. The gating strategy for Foxp3^+^Helios^+^ and Foxp3^+^CD25^+^ from CD4^+^ T cells was shown in Fig. 1a. In pairwise comparisons, the frequency of Foxp3^+^Helios^+^ is significantly higher than those of Foxp3^+^CD25^+^ in both HIV-1-infected individuals and HC (Fig. 1b).

In CHI&ART- patients, the frequencies of Foxp3^+^Helios^+^ Treg cells, memory Foxp3^+^Helios^+^CD45RO^+^ Treg cells and naïve Foxp3^+^Helios^+^CD45RA^+^ Treg cells were significantly higher than those of HC, AHI and CHI&ART+ (Fig. 1c, Fig. 1d, and Fig. 1e). In acute HIV-1-infected patients, the frequency of naïve Foxp3^+^Helios^+^CD45RA^+^ Treg cells was significantly higher than that of HC and CHI&ART+, whereas it was significantly lower than that of CHI&ART- (Fig. 1e).

The frequency of Foxp3^+^Helios^+^CD45RA^+^ Treg cells was inversely correlated with CD4 T-cell counts and CD4/CD8 ratio during acute and chronic HIV-1 infection (Fig. 1f and Fig. 1g). There was no correlation between viral loads and the levels of Foxp3^+^Helios^+^ (*r* = − 0.07, *p* = 0.54), Foxp3^+^Helios^+^CD45RO^+^ (*r* = 0.05, *p* = 0.71), and Foxp3^+^Helios^+^CD45RA^+^ Treg cells (*r* = − 0.18, *p* = 0.17) in AHI and CHI&ART- patients (Fig. 1h).

### PD-1 expression on three monocyte subsets in HIV-1-infected individuals

**Fig. 2:**
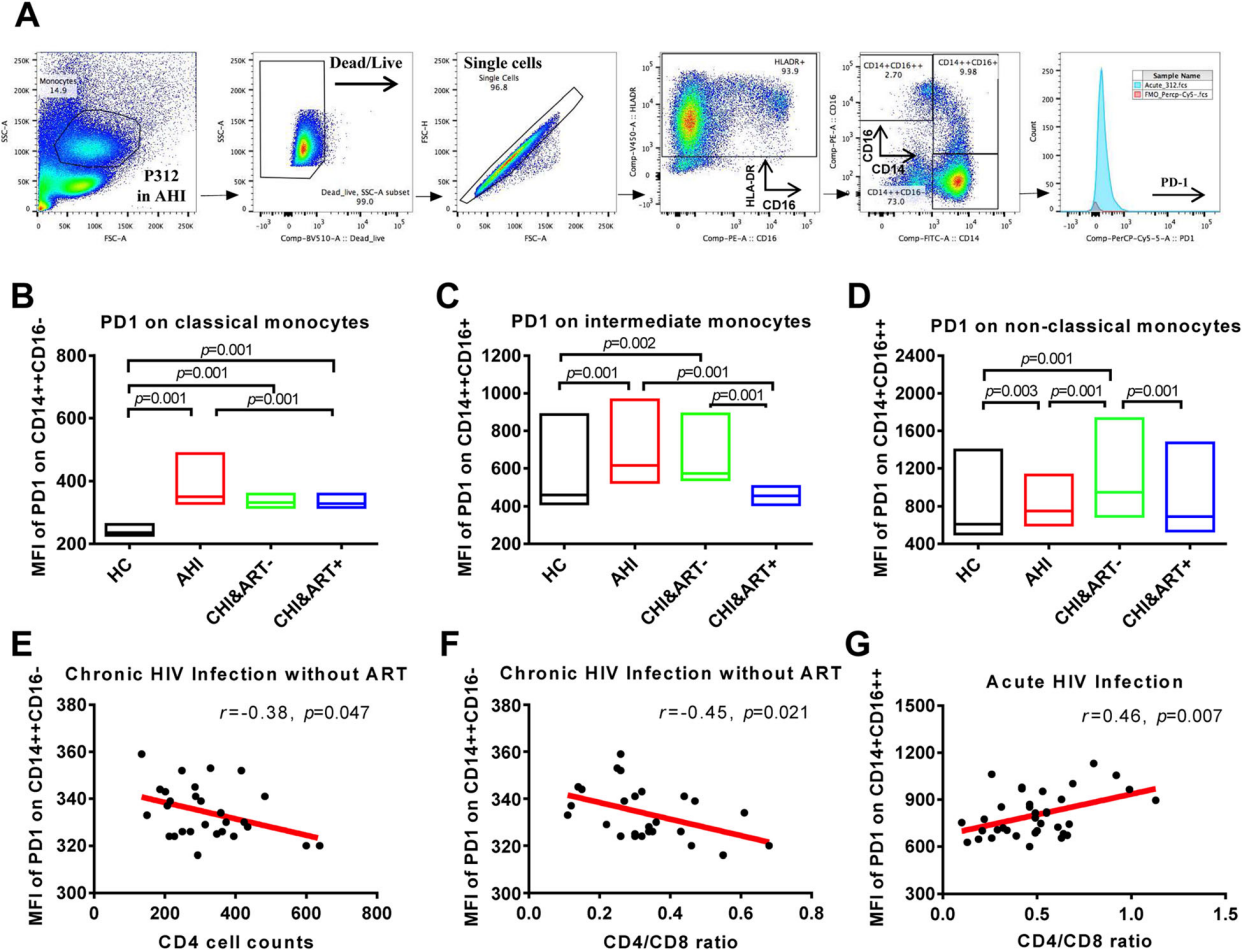
Perturbations of PD-1 expression on the three monocyte subsets in HIV-1-infected patients. The gating strategy for PD-1 expression on monocyte subsets is indicated (**a**). The MFI of PD-1 on CD14^++^CD16^−^ (**b**), CD14^++^CD16^+^ (**c**), and CD14^+^CD16^++^ (**d**) monocytes were analyzed by flow cytometry in HC, AHI, CHI&ART-, and CHI&ART+ patients. Correlations between the MFI of surface PD-1 on CD14^++^CD16^−^ and CD4 T-cell counts (**e**) as well as the CD4/CD8 ratio (**f**) in chronic HIV-1-infected cART-naïve patients. Correlation between MFI of surface PD-1 on CD14^+^CD16^++^ and the CD4/CD8 ratio (**g**) in acute HIV-1-infected patients. The box marks the min and max values, and horizontal lines depict median values in (**b**), (**c**) and (**d**). All *p* values were calculated using an ANOVA, Student’s t-test or the Mann-Whitney U test, and Spearman’s correlation coefficient

The gating strategy for PD-1 expression on monocyte subsets is shown in Fig. 2a. PD-1 expression of three monocyte subsets of AHI and CHI&ART- patients was significantly higher than that of HC (Fig. 2b, Fig. 2c and Fig. 2d). In acute HIV-1-infected patients and CHI&ART- patients, the mean fluorescence intensity (MFI) of PD-1 expression on intermediate CD14^++^CD16^+^ monocytes was significantly higher than those of HC and CHI&ART+ (Fig. 2c). The MFI of PD-1 expression on non-classical CD14^+^CD16^++^ monocytes of CHI&ART- is significantly higher than that of HC, AHI and CHI&ART+ patients (Fig. 2d).

In CHI&ART- patients, the expression of PD-1 on classical CD14^++^CD16^−^ monocytes is inversely correlated with CD4 T-cell counts (Fig. 2e) and CD4/CD8 ratio (Fig. 2f). The expression of PD-1 on non-classical CD14^+^CD16^++^ monocytes is positively correlated with the CD4/CD8 ratio during acute HIV-1 infection (Fig. 2g).

### The correlation between Foxp3^+^Helios^+^Treg cells and monocyte subsets and their PD-1 expression during acute HIV-1 infection

**Fig. 3:**
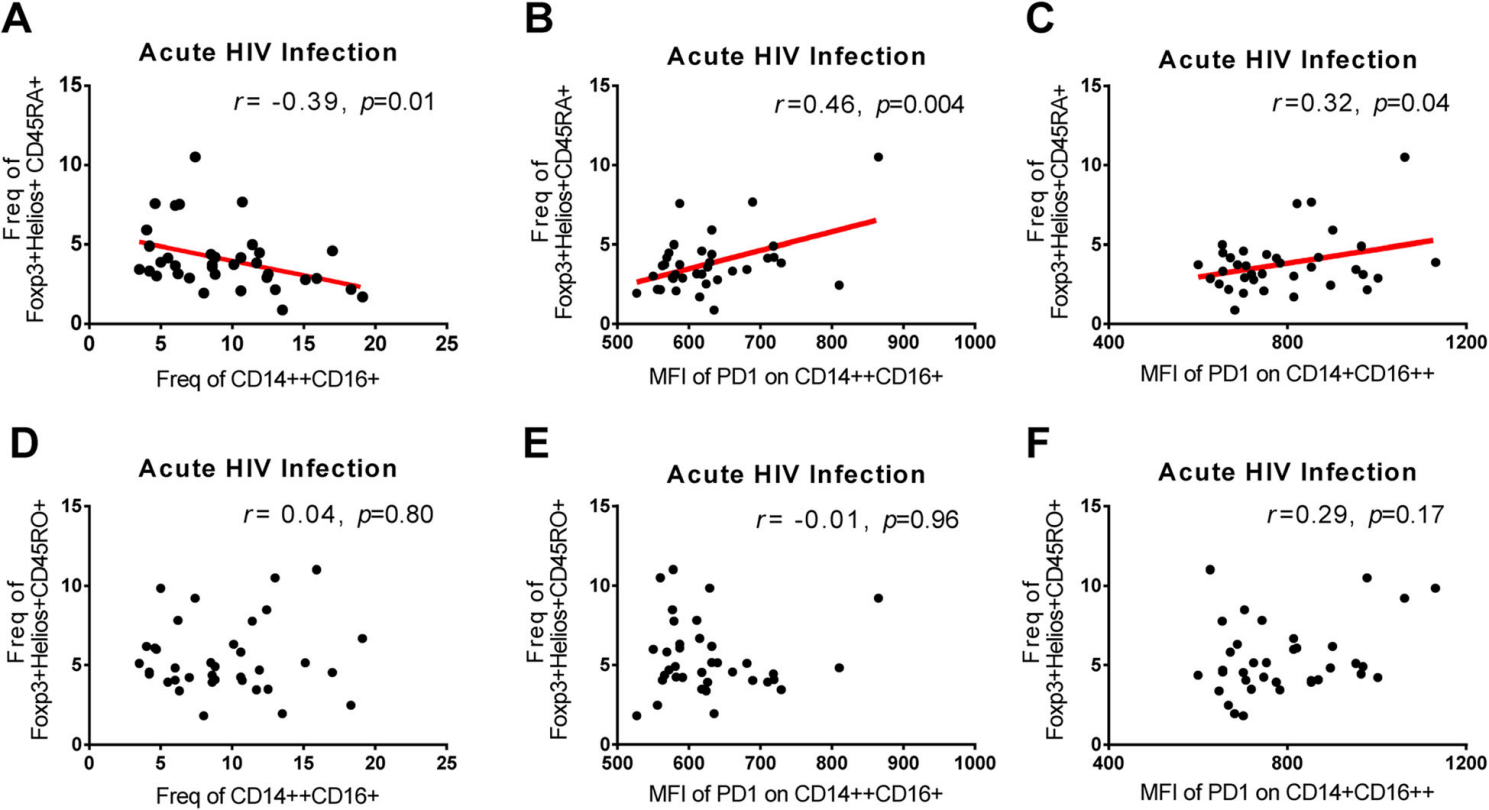
Correlations between the frequency of Foxp3^+^Helios^+^ Treg cells and monocyte subsets and their PD-1 expression in HIV-1-infected individuals. Correlation between the frequency of Foxp3^+^Helios^+^CD45RA^+^ Treg cells and CD14^++^CD16^+^ monocyte subsets (**a**), PD-1 density on CD14^++^CD16^+^ monocytes (**b**), and PD-1 density on CD14^+^CD16^++^ monocytes (**c**) during acute HIV-1 infection. Correlation between the frequency of Foxp3^+^Helios^+^CD45RO^+^ Treg cells and CD14^++^CD16^+^ monocyte subsets (**d**), PD-1 density on CD14^++^CD16^+^ monocytes (**e**), and PD-1 density on CD14^+^CD16^++^ monocytes (**f**) during acute HIV-1 infection. Correlations were analyzed by calculating Spearman’s correlation coefficient

The correlation between monocyte subsets and their PD-1 expression and Foxp3^+^Helios^+^ Treg cells is shown in Fig. 3. During acute HIV-1 infection, the frequency of Foxp3^+^Helios^+^CD45RA^+^ Treg cells was inversely correlated with the frequency of intermediate CD14^++^CD16^+^ monocytes, whereas it was positively correlated with the PD-1 density on intermediate CD14^++^CD16^+^ monocytes (Fig. 3a and Fig. 3b). In addition, the PD-1 density on non-classical CD14^+^CD16^++^ monocytes is positively correlated with the frequency of Foxp3^+^Helios^+^CD45RA^+^ Treg cells during acute HIV-1 infection (Fig. 3c). There was no correlation between the frequency of Foxp3^+^Helios^+^CD45RO^+^ Treg cells and the levels of CD14^++^CD16^+^ monocyte subsets, PD-1 expression CD14^++^CD16^+^ monocyte, and PD-1 expression CD14^+^CD16^++^ monocyte (Fig. 3d-f).

## Discussion

The specific roles of three monocyte subsets and their PD-1 expression on Treg cell differentiation have remained poorly understood in acute HIV-1-infected patients. In the present study, we characterized the perturbations of Foxp3^+^Helios^+^ Treg cells during HIV-1 infection. We found that in acute HIV-1-infected patients, the frequency of Foxp3^+^Helios^+^CD45RA^+^ Treg cells was inversely correlated with the frequency of intermediate CD14^++^CD16^+^ monocyte subsets, whereas it was positively correlated with PD-1 expression on intermediate CD14^++^CD16^+^ monocyte subsets.

Foxp3^+^Helios^+^ Treg cells regulate the immune response due to suppressive abilities. Monocytes are heterogeneous with subset-specific phenotypes and functions, and have been reported to control Treg cell differentiation. PD-1 signaling may also occur independently of T-cell or B-cell antigen receptor signaling, and the expression of PD-1 on monocytes could modulate immune responses. In the study, monocyte subsets and their PD-1 expression were found to have varying impacts on Treg-cell differentiation in HIV-1-infected patients. Therefore, understanding how monocyte subsets and their PD-1 expression influence Treg cells may aid in boosting or preventing pathological responses during HIV-1 infection.

The perturbations of Treg cells during AHI remain controversial, which is partially because of the gating strategy and the lack of consensus phenotypic markers that define Treg cells [21, 22]. In our study, we found that in acute HIV-1-infected patients, the frequency of Foxp3^+^Helios^+^CD45RA^+^ Treg cells was significantly higher than those of both HC and HIV-1-infected patients after cART. There are several possible causes for the Treg cell expansion, including enhanced Treg cell generation and increased cell survival [23, 24]. Previous studies have shown that there is an inverse correlation between Treg cells frequency and CD4 T-cell counts [25], which is consistent with our findings. In our study, the frequency of Foxp3^+^Helios^+^CD45RA^+^ Treg cells in acute HIV-1-infected patients was significantly higher than those of HC and CHI&ART+, whereas CD4 T-cell counts of acute HIV-1-infected patients were lower than that of HC and CHI&ART+. Reduced Treg cell numbers are associated with a reduced suppression capacity of CD8^+^ T cells, NK cells, and other immune cells [26]. Systemic immune activation is a consequence of HIV-1 infection, and cART produces profound suppression of HIV replication, but fails to eliminate chronic immune activation completely. An increased proportion of Treg cells during chronic HIV-1 infection may be beneficial for controlling persistent hyperactivity [27]. Due to the beneficial or detrimental roles of Tregs in HIV-1 infection, different therapeutic strategies will be considered to enhance the immune response or to downregulate global hyperactivity by pharmacologic manipulation of Tregs or transferring adoptive cells.

Monocytes are generally regarded as precursors of macrophages and dendritic cells (DCs), and DCs have the capacity to expand antigen-specific Tregs [28]. The CD16^+^ monocyte subset was positively correlated with IFN^−^ CD4^+^ T cells, but negatively correlated with CD4^+^CD25^hi^Foxp3^+^ Treg cells in immune thrombocytopenia [29]. In this study, we found that the frequency of intermediate CD14^++^CD16^+^ monocytes was inversely correlated with the frequency of Foxp3^+^Helios^+^CD45RA^+^ Treg cells in acute and chronic HIV-1-infected patients. Defining the influence of monocytes on Tregs will enable more precise targeting of immune cells to enhance defense against HIV-1 infection.

PD-1 and its related pathways are considered a central regulator of T cell exhaustion [30], but the function of PD-1 expression on APCs requires further clarification. During *Listeria monocytogenes* (LM) infection, PD-1 can be induced on splenic DCs, and PD-1 on DCs negatively regulates IL-12 and TNF-α production and inhibits innate immune responses [31]. PD-1 expression on monocytes/macrophages in patients with sepsis is higher than that in HC. Moreover, PD-1 expression on monocytes is associated with cellular dysfunction [32]. PD-1 signaling inhibits T-cell activation, but can promote induced regulatory T-cell development [33]. In this study, we found that the expression of PD-1 on both intermediate CD14^++^CD16^+^ and non-classical CD14^+^CD16^++^ monocyte subsets was positively correlated with the frequency of Foxp3^+^Helios^+^CD45RA^+^ Treg cells in HIV-1-infected patients. PD-1:PD-L1 interactions can limit effector T-cell responses, and induce regulatory T cells. Significant clinical activity has been shown in a variety of cancers by targeted therapy against PD-1/PD-L1 [34]. Therapeutic targeting of the PD-1:PD-L1 pathway could be beneficial for the enhancement of immune responses against viral pathogens.

In this study, an in vitro stimulation system was used, in which PBMCs were stimulated with PMA/ionomycin. Monocyte and T-cell coculture systems and ex vivo or in vitro experiments will be performed in future studies to investigate the roles of monocyte subsets and their PD-1 expression on Treg differentiation. Monocytes are generally regarded as precursors of macrophages and DCs; therefore, it is of interest to characterize the modulation of CD4^+^ Tregs by monocyte-derived DCs [35, 36].

## Conclusions

In this study, the perturbations of Foxp3^+^Helios^+^ Treg cells were characterized, and the association between monocyte subsets and their PD-1 expression and Foxp3^+^Helios^+^ Treg cells was evaluated during acute HIV-1 infection. Our observations provide new evidence for the roles of Foxp3^+^Helios^+^ Treg cells and PD-1 expression on monocyte subsets in HIV pathogenesis. Understanding the effects of monocyte subsets and their PD-1 expression on Foxp3^+^Helios^+^ Treg cells may be helpful for developing different therapeutic strategies to manipulate immune responses against HIV-1 infection.

## Data Availability

The data and materials in the study are available from the corresponding author on reasonable request.

## Abbreviations

AHI: acute HIV-1 infection
APC: antigen-presenting cells
cART: combined antiretroviral therapy
CHI&ART-: chronic HIV-1-infected cART-naïve patients
CHI&ART+: chronic HIV-1-infected patients with undetectable viral load after cART
HC: healthy controls
MFI: mean fluorescence intensity
MSM: men who have sex with men
PD-1: programmed cell death protein 1;
Treg: regulatory T
